# Detection of Management-Frames-Based Denial-of-Service Attack in Wireless LAN Network Using Artificial Neural Network

**DOI:** 10.3390/s23052663

**Published:** 2023-02-28

**Authors:** Abdallah Elhigazi Abdallah, Mosab Hamdan, Mohammed S. M. Gismalla, Ashraf Osman Ibrahim, Nouf Saleh Aljurayban, Wamda Nagmeldin, Mutaz H. H. Khairi

**Affiliations:** 1 Faculty of Computer Science, Future University, Khartoum 10553, Sudan; 2 Faculty of Computing and Informatics, Universiti Malaysia Sabah, Kota Kinabalu 88400, Malaysia; 3 Department of Computer Science, University of São Paulo, São Paulo 05508-090, Brazil; 4 Faculty of Electrical Engineering, Universiti Teknologi Malaysia, Skudai 81310, Malaysia; 5 Advanced Machine Intelligence Research Group, Universiti Malaysia Sabah, Kota Kinabalu 88400, Malaysia; 6 Department of Information Systems, College of Computer Engineering and Sciences, Prince Sattam bin Abdulaziz University, AL-Kharj 11942, Saudi Arabia; 7 Faculty of Engineering, Future University, Khartoum 10553, Sudan

**Keywords:** artificial neural network, media access control (MAC), wireless local area network (WLAN), denial of service (DoS)

## Abstract

Wireless Local Area Networks (WLANs) have become an increasingly popular mode of communication and networking, with a wide range of applications in various fields. However, the increasing popularity of WLANs has also led to an increase in security threats, including denial of service (DoS) attacks. In this study, management-frames-based DoS attacks, in which the attacker floods the network with management frames, are particularly concerning as they can cause widespread disruptions in the network. Attacks known as denial of service (DoS) can target wireless LANs. None of the wireless security mechanisms in use today contemplate defence against them. At the MAC layer, there are multiple vulnerabilities that can be exploited to launch DoS attacks. This paper focuses on designing and developing an artificial neural network (NN) scheme for detecting management-frames-based DoS attacks. The proposed scheme aims to effectively detect fake de-authentication/disassociation frames and improve network performance by avoiding communication interruption caused by such attacks. The proposed NN scheme leverages machine learning techniques to analyse patterns and features in the management frames exchanged between wireless devices. By training the NN, the system can learn to accurately detect potential DoS attacks. This approach offers a more sophisticated and effective solution to the problem of DoS attacks in wireless LANs and has the potential to significantly enhance the security and reliability of these networks. According to the experimental results, the proposed technique exhibits higher effectiveness in detection compared to existing methods, as evidenced by a significantly increased true positive rate and a decreased false positive rate.

## 1. Introduction

Regarding availability, denial of service (DoS) attacks represent a severe threat. They restrict access to resources or services for the targeted user. The DoS attack’s effects are exceedingly dangerous. Recent denial of service assaults against small and medium-sized businesses and government websites have had a significant social impact [1]. DoS attacks are simple to carry out, especially in the wireless domain, due to the broadcast environment of wireless networks [2,3].

DoS attack aims to make network resources inaccessible to other authorised users [4,5]. While data is changed or stolen in different types of assaults, a DoS attack seeks to impede or exhaust system resources for other users. The assailants have a variety of objectives; he does it for ideology, money, or just plain enjoyment. In a DoS attack, the initial stage is to produce large amounts of malicious traffic [6,7] and send that traffic in the victim network’s or resources’ direction, utilising the entire target’s computing resources. As a result, authorised users are unable to access system resources [8]. A DoS attack can take down a wireless local area network (WLAN) [1,9]. Confidentiality, integrity, and availability are the three protections of each packet broadcast in the air that we focus on most when discussing wireless security. Different protocols, including wired equivalent privacy (WEP), wi-fi protected access (WPA), and WPA2 are primarily responsible for managing confidentiality and integrity in WPA2. However, DoS attacks are a threat that WLANs are still susceptible to [10]. The 802.11i places less emphasis on availability and more on maintaining integrity and privacy [11,12]. In light of this, DoS attacks continue to be a severe 802.11i vulnerability despite the robust security network association (RSNA) [13].

The motivation behind using NN in this research is that NNs are highly flexible and can analyse incomplete or partial data. However, the parallel processing feature of NNs and WLAN traffic can generate a significant amount of overhead on the monitoring STA, which can affect its performance and sometimes lead to denial-of-service (DoS) attacks. In addition, we used NNs because of their ability to learn and adapt to new data, recognize patterns in data, and update their detection capabilities accordingly. This can be difficult for humans or traditional machine-learning algorithms to achieve. NNs can improve detection accuracy and reduce false alarms. Additionally, NNs can be trained to identify patterns in network traffic that are indicative of various types of attacks, including DoS attacks. Automated detection using NNs can save time and effort compared to manual detection and enable a quick response to attacks.

Two main tasks are carried out in this paper. Firstly, the Enhanced De-authentication and Disassociation Detection Scheme (ED3S) was proposed to improve the De-authentication and Disassociation Detection Scheme (D3S). The new scheme decreases the false alarm rate and increases the detection accuracy of the original D3S scheme. The Enhanced De-authentication and Disassociation Detection Scheme (ED3S) employed features generated by the artificial neural network to build the detection model. Secondly, the Enhanced Scheme for Detecting Management-Frames-Based DoS Attacks was designed and developed by integrating our published model Data-Centric Resource Exhaustion Attack Detection Scheme (READS) with D3S. The ED3S scheme aims to improve D3S detection performance by increasing detection accuracy under resource exhaustion attacks and masquerading attackers. The main idea is to develop an intelligent feature through which fraudulent nodes can be identified more easily. These features are more representative of such attacks than manually crafted ones. The concept is to use the distance between the message ranks to the cluster centroid in the model developed in our previous work [14]. This new feature is called the Packet Spoofing Score (PSS), which was obtained by integrating the model proposed in our previous work [15].

The remainder of the article is constructed as follows: Section 2 is a related work section. In contrast, Section 3 presents the proposed enhanced ED3S scheme. In addition, Section 4 describes the details of the design and implementation of the proposed scheme. The experimental setup has been detailed in Section 5, while the proposed scheme’s evaluation considers the experimental results discussed along with the comparison with related works. Lastly, Section 6 shows the conclusion of the paper.

## 2. Related Works

Chen et al. [16,17] have independently suggested using the k-means clustering algorithm to find rogue access points that are faking signals or frames. This research was predicated on the idea that, in the absence of an additional rogue access point (AP), the sequence of the last Received Signal Strength Indicator (RSSI) values received from that AP would have little oscillations around the mean (i.e., an Evil Twin). When employing the k-means algorithm to divide the components of a received RSSI sequence into two clusters, there would be little space between the two computed centroids if there weren’t an Evil Twin (i.e., lesser than a threshold value). In addition, a significant gap between the centroids of the two newly formed clusters would suggest the presence of an Evil Twin AP with a distinctive RSSI distribution. However, because their method lacks offline learning (i.e., a previously learned model of what constitutes a legal distribution), for detection to take place, communication between the authentic node and the MAC address spoofer must take place often.

In reference [18], they described a method for identifying probe request attacks by classifying real-world WLAN data frames from a station (STA) using a neural network (NN) classifier. Signal strength, sequence number, frame sub-type, and delta time were the four variables used to train the supervised feed-forward NN classifier to distinguish between genuine and malicious frames. According to experimental results, the NN-based model accurately detects probe request assaults and distinguishably does so when they are still in their early stages. Furthermore, the current method of separating legitimate frames from corrupted ones using actual traffic data for NN is labour-intensive and manual. Sheng et al. [19] studied how antenna diversity affected the detection of spoofing and device fingerprinting using RSSI in 802.11 access points. They showed how the antenna variety allowed by the 802.11 standard causes the RSSI data from a stationary receiver obtained at a stationary emitter to result in a blend of two gaussian distributions.

For each wireless node and access point pair in the network, they constructed a Gaussian mixture model. On the sequence of the most recent RSSI received at each access point from a specific MAC address, they ran a log-likelihood ratio test. If the ratio test fails by more than n Gaussian mixture models, where n is less than the total number of networked access points and must be determined empirically, the transmitting node is taken to be a fake. However, a competitor may change its transmission power to evade detection by this model by using easily accessible off-the-shelf hacking tools.

Gonzales et al. [20] have created a cutting-edge method called context-leashing for spotting open Evil Twin access points. They have stated that widely accessible access points, such as those found in chain coffee shops (like Starbucks), frequently lack authentication, and share the exact service set identification (SSID) across numerous locations; this allows adversaries to fake such SSIDs and persuade customers to connect to the malicious access point. Moreover, reference [15] suggests an NN-based MAC spoofing detection method. Using sparse, noisy, imperfect, and nonlinear data sources, they have the capacity to detect and categorise network behaviour. Another benefit of NNs is their rapid processing of enormous amounts of data. A prototype implementation was used to verify the proposed method. The validation results showed that the suggested method successfully achieves a high result for mutually real and fake MAC addresses.

The defence against the Evil Twin APs offered in [21] is predicated on employing a context-leashing engine. Upon affiliation with a publicly accessible access point, the context-leashing engine would compile a list of context $Ci=\left(c1,\;r1\right),\;\left(cn,rn\right)$. This list contains a list of all accessible SSIDs that are reachable at the moment of association with a given SSID in the environment, as indicated by the letters c j and r j, respectively. A linked list is prepared for each connected SSID. A new context list is built and compared with the one previously kept for any future reassociation with a specific SSID. The connected SSID is considered an Evil Twin. The connection should be severed if there is no significant (empirically determined) link between the accessible nearby SSIDs, their average RSSI values, and the historical context list. The fundamental flaw in their approach is that it assumes that the list of SSIDs in a specific geolocation would stay essentially the same over time, which is untrue given the tethering capabilities of modern smartphones.

Based on our survey of related studies, it appears that neural networks (NN) offer a high degree of flexibility and are capable of analysing incomplete or partial data. However, it is important to note that utilizing WLAN traffic and the parallel processing feature of NNs can result in a substantial amount of overhead on the monitoring station, which may impact its performance and even cause a denial of service (DoS) in some cases.

## 3. Proposed Scheme

**Figure 1:**
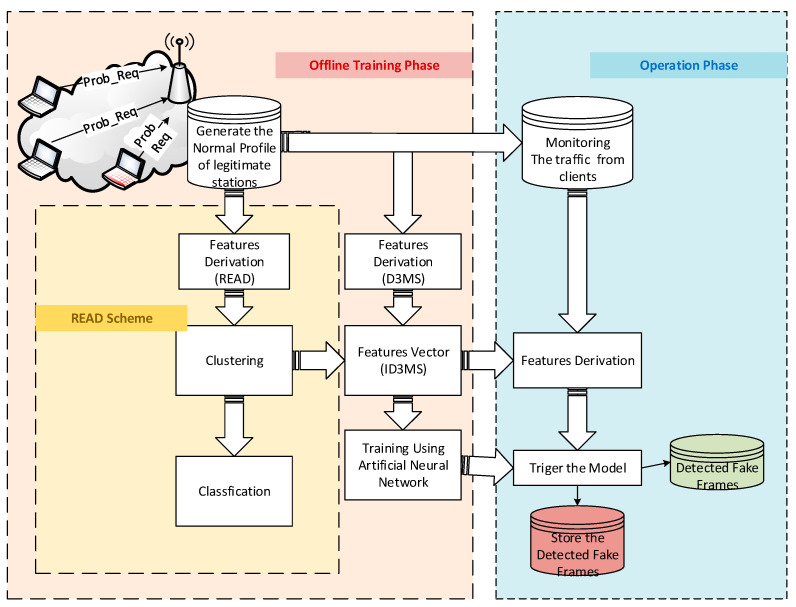
The online and offline phases of the scheme.

The proposed ED3S Scheme aims to effectively detect fake de-authentication/disassociation frames and improve network performance by avoiding communication interruption by such attacks. The proposed scheme integrates the model proposed and developed in [14] with the scheme proposed and developed in [15]. The integration enables the scheme to obtain better features that accurately represent fake messages. Figure 1 shows the proposed scheme.

## 4. Details of the Scheme

The proposed approach includes two essential parts, offline training, and online operation, as seen in Figure 1. The model is trained, the data is collected, and the distinguishing features are determined during the offline training phase. The scheme’s capacity for detection is examined during the online procedure.

### 4.1. Offline Training Phase

In the offline phase, three steps were conducted to build the classifier. The first step is the collection of the raw data from the wireless WLAN scenario. The second step is to derive the features representing the station’s everyday activities. Then, in the third phase, the neural network classifier is built.

#### 4.1.1. Data Gathering

The classification model used in this research was built using a realistic dataset collected from a wireless local area network scenario. The scenario was set up with a wireless access point and six stations with different operating systems including Windows, macOS, and Android. These stations were used to represent normal de-authentication and dis-association behaviour, with both static and mobile stations included in the dataset.

To collect the data, one station’s network interface with MacOS was put into monitoring mode, allowing for the recording of the connected devices’ traffic behaviour. The resulting dataset was then used to generate features that represent normal activities, which were later used to train the NN model. This dataset, along with the use of an NN, helped ensure that the model was representative of real-world network behaviour and capable of accurately detecting management-frames-based DoS attacks. After the NN model was trained, it was tested to evaluate its performance in detecting these attacks.

#### 4.1.2. Derivation of Features

The aspects that were taken out during standard de-authentication and dis-association processes are explained in this section. These characteristics were employed by the proposed Scheme to differentiate between fake and real disassociation and de-authentication frames:

1. Time of last Authentication/Association (TLA): TLA is the amount of time that passes between a request for de-authentication or disassociation and the answer to the most recent request for authentication or association.
2. Time to last data packet received (TLD): TLD is the interval between the de-authentication/disassociation request and the preceding data packet.
3. Data Rate at De-authentication/Disassociation Frames Request (D.R.): The D.R. is the typical station data rate computed from the most recent time frame prior to the re-receipt of the de-authentication/disassociation request.
4. The signal strength of the de-authentication/disassociation request frame is represented by the RSSI value of the de-authentication/disassociation frame (RSSI).
5. Sequence Jumping Distance (SJD): the distance between the most recent and preceding sequence numbers.
6. Packet Spoofing Score (PSS): The distance between the message rank and the cluster centroid.

#### 4.1.3. Dataset Replication and Attack Simulation

Because the real dataset has few samples, the replication of the dataset is a common procedure before training so that the variability associated with normal user behaviour can be estimated. To generate more samples of the real dataset, the data samples were replicated to represent different possible behaviour of the users. The replicated samples have been randomly generated from a normal profile of normal behaviour in the real dataset. The dataset samples were replicated to create more samples with different behaviours.

An attack model that might involve de-authentication and disassociation was used to simulate the attacker’s data. The attacker begins by observing the flow of traffic. Then, among the stations already associated with the access point, the targeted station is located. To impersonate the victim, the attacker changes his own MAC address and adjusts it to resemble the MAC address of the station being targeted. The attacker then approaches the access point with a false de-authentication/disassociation request. By employing the model created in the next section, access point evaluates the message’s legitimacy.

#### 4.1.4. Model Construction

To build the scheme, a NN was employed, as it can represent complex nonlinear hypotheses that a linear classifier model cannot describe. Such NNs act as an intelligent agent whose purpose is to identify the underlying patterns in abnormal and normal management frames by monitoring and analysing the historical traffic data recorded during normal operations [22]. To build the model, a feed-forward neural network with a backpropagation algorithm was used to train a classifier to differentiate between genuine and fake frames.

The training involved a three-layered neural network with hidden input and output layers on each side. Different numbers of neurons were employed in each layer. Six neurons make up the input layer, and each one represents a feature. A second neuron was added to the input layer as a bias if a zero vector was introduced into the input layer, which can make the training process more flexible and accurate. One neuron serving as the class label is present in the output layer. The correct number of hidden layers must be determined to avoid overfitting neural networks. As a result, the formula in the equation was used to estimate the number of neurons in the hidden layer, as shown in Equation (1). Six neurons were consequently selected for the hidden layer.

(1) $$N_{h}=\frac{N_{s}}{\left(\alpha \times \left(N_{i}+N_{o}\right)\right)}\;$$

$N_{s}$ is the number of training dataset parts; *N_i_* is the number of input layer neurons, *N_o_* is the number of output layer neurons, and a scaling factor has been heuristically chosen through trial and error. Many experts advise keeping the range of values between 5 and 10 to avoid overfitting.

It initialises the neural network’s weights at random with small amounts close to zero. A backpropagation approach called “gradient descent” adjusts the network weights and biases them towards the performance function’s most significant deviation. This algorithm’s first iteration can be expressed as follows:

(2) $$\theta_{k+1}=\theta_{k}-\propto_{k}g_{k}$$

where $\theta_{k}$ are a vector’s current weights and biases, $g_{k}$ is the current gradient while $\propto_{k}$ is the learning rate.

### 4.2. Online Operation

**Figure 2:**
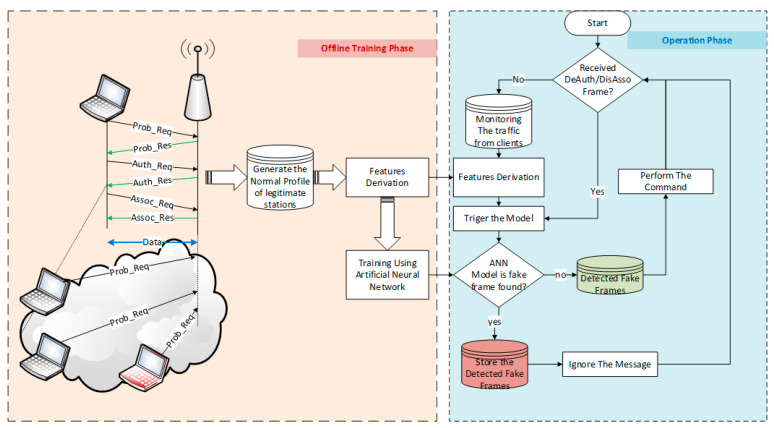
Online and offline stages.

After training the model, it becomes ready to detect and mitigate attacks. The flowchart of the detection process is illustrated in Figure 2.

1. The access point listens to the benign stations’ upcoming association and authentication requests. The stations could be stationary such as PCs, or mobile, such as laptops and smartphones.
2. Upon receiving any association and authentication request from a client station, the access point executes a request and responds to the client through an authentication or association response.
3. The access point monitors the traffic activities of every client. It keeps their details in a temporary buffer for a period of time that is determined when the access point receives a de-authentication or disassociation request from the client.
4. Upon receiving any de-authentication or de-authentication/disassociation request from a client station, the access point derives the feature vector according to the procedure described in Section 3.
5. The access point triggers the neural network classification model to classify the requesting station based on the derived vector. The classifier predicts the vector label and suggests the class.
6. According to the class label, the access point chooses to execute or drop the request from the station.

## 5. Experimental Setup

There are two main procedures for the experiment described in this section. Firstly, the normal traffic was captured to create the normal profile. Secondly, the attack activities are simulated and inserted into the data samples. More details of these procedures are provided in the following:

### 5.1. The Traffic Capturing

Data was gathered utilizing actual experiments because there weren’t enough labelled datasets available that had the attacker’s ground truth. The following was recorded as the typical traffic behaviour: The AP used a TP-LINK DG834GT wireless N router with a MAC address of F81A67DF22B2. Because the access point is configured for open-access wireless LAN, any wireless station can connect to it. There were five user stations connected to the access point. The wireless traffic was recorded using a single station. The recording device was a MacBook Pro running OS X EI Caption v10.11.6. This capturing device’s wireless adapter was configured to monitor mode to record all frame types, including control and management frames. The monitoring mode can sniff the MAC layer traffic on the AP working channel. The monitoring mode, as opposed to the promiscuous mode, enables the adapter to sniff airborne communication without associating with the access point. The network traffic was recorded using Wireshark v2.6.5.

**Figure 3:**
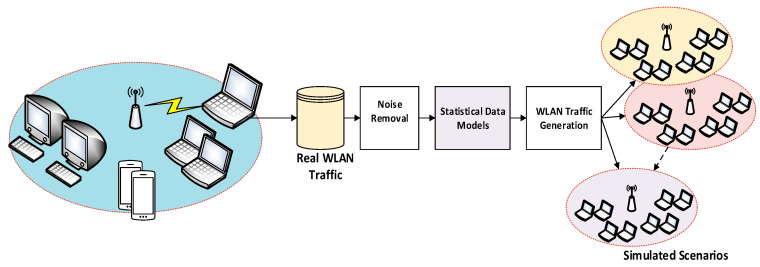
Capturing and simulating WLAN scenarios.

Two main replication processes were carried out to generalize the findings across various datasets, given that only five stations were used for data gathering. The data was first amassed over time to increase the number of requests for de-authentication and disassociation. Second, to mimic WLAN behaviour during normal operation, computer-generated data was collected. The usual station and access point behaviour in response to the measured traffic metrics was modelled using statistical techniques. To simulate 100 workstations, the client and access point paradigm was used in MATLAB. The simulation used the data that was gathered from the real world. The conceptual organization of the dataset collection scenario is shown in Figure 3.

### 5.2. DE-Authentication/Disassociation Attack Simulation

The basic de-authentication/disassociation attack is summarised below:

1. By keeping an eye on the network traffic for a while, the attacker locates the victims among the clients connected to the AP.
2. The attacker modifies its MAC address to precisely match the victim’s station.
3. The perpetrator launches a phoney de-authentication/disassociation attack.
4. The AP responds by executing the request from the MAC address of the request if it is not protected.

By randomly sending fictitious de-authentication/disassociation signals, the attacker’s data are simulated from a formation attack step. By training the categorization model after receiving the de-authentication/disassociation messages, the model is put to the test. If the message were accurate, the pre-trained model would probably confirm the generated feature vector and assign the expected label. If the message is created by an attacker, the derived feature vector will diverge from the normal vector, and the classification outcome will most likely favour the abnormal class.

### 5.3. Performance Evaluation

The accuracy, false-positive rate (FPR), false-negative rate (FNR), and F-score (F-measure) were the main metrics for evaluating the scheme performance. FPR and FNR are common evaluation metrics for validating the effectiveness of the tested attack scenarios. Given that the number of fake messages is not necessarily equal to the number of genuine messages, the F-score is an important performance evaluation metric in this situation. F-score is the harmonic mean between precision and recall that evaluates how the system performs with respect to the trade off between FPR and FNR.

#### 5.3.1. Analysis and Discussion of the Results

**Figure 4:**
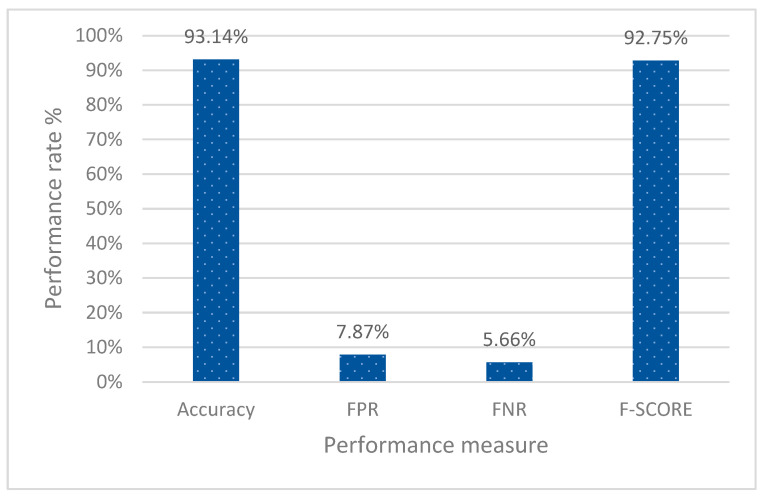
The effectiveness of the proposed scheme.

The experimental results are reported and analysed in terms of the following performance metrics; detection accuracy, detection rate, FPR, FNR, and F-measure. Figure 4 shows the average results obtained by applying the proposed scheme during the operational mode. The results have been averaged for the four testing scenarios simulated with randomness in both attack and normal behaviour.

As shown in Figure 4, the scheme achieved 93.83% detection accuracy. The active FPR was 7.73%, while the FNR was 4.49%. The overall performance of the model in terms of F-score was 93.40%. These results suggest that 7.73% of genuine messages will be dropped and will not be executed by the AP. This implies that the limited resources of the access point will be unnecessarily reserved for a station that is no longer connected to the network. Another finding is that the attack success rate will reach 4.49%, meaning that from every 100 attack attempts, only about five will be successful. A detailed investigation has been carried out to validate the proposed model’s performance.

#### 5.3.2. Comparison and Result Analysis

**Figure 5:**
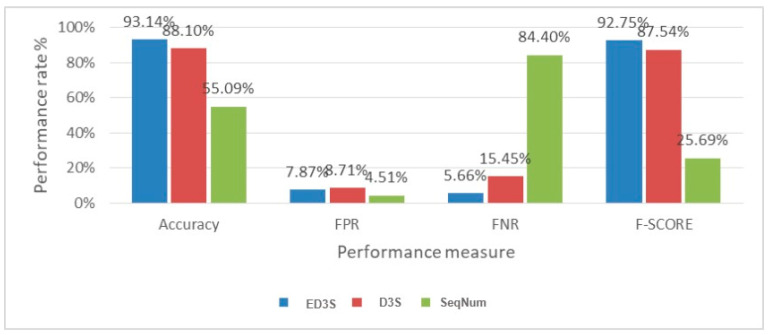
Comparison between ED3S, D3S, and SeqNum.

To demonstrate the enhancement provided by the proposed ED3S scheme [15], a comparison was performed in terms of overall accuracy, FPR, FNR, and F-measure, as well as the associated SeqNum-based scheme. Table 1 provides the numerical outcomes of the evaluation metrics for four scenarios. Figure 5 compares the performance of the comparable SeqNum Based scheme to that of the proposed scheme.

Table 1 and Figure 5 show that the proposed scheme ED3S achieved the highest accuracy, 93.14%, compared to 88.10% for the D3S and 55.09% for SeqNum. This is because ED3S uses PSS. Furthermore, PSS was used to pre-knowledge the node behaviour, thus increasing the detection rate.

Contrary to ED3S, D3S uses handcrafted features that could not completely represent attack patterns. Meanwhile, the SeqNum-based scheme considers only the sequence number, which can easily be evaded, as the main factor for distinguishing fake messages. The proposed scheme, in contrast, has been trained using broader, more innovative features that accurately depict the typical flow of the de-authentication/disassociation processes. The proposed scheme increased the SeqNum-based scheme’s FPR from 7.87% to 8.71% in terms of FPR reduction. However, it is lower than that archived by D^3^S. Although there are 3.36% increases in the FPR compared to the SeqNum scheme, the high FNR of the SeqNum-based scheme indicates its ineffectiveness. The findings indicate that the SeqNum-based scheme miss-classifies 84.40% of fake messages. That is why it advances in order to expose the network to an attack. Comparing the SeqNum-based scheme’s FPR and FNR demonstrates that it could not achieve a fair trade off between the two rates. This explains why the SeqNum-based scheme’s false positive rate is lower than that of both ED^3^S and D^3^S. In terms of overall performance, the proposed ED3S Scheme reached the highest F-Score (92.25%) compared to 87.54% and 25.69% by D3S and SeqNum, respectively.

**Figure 6:**
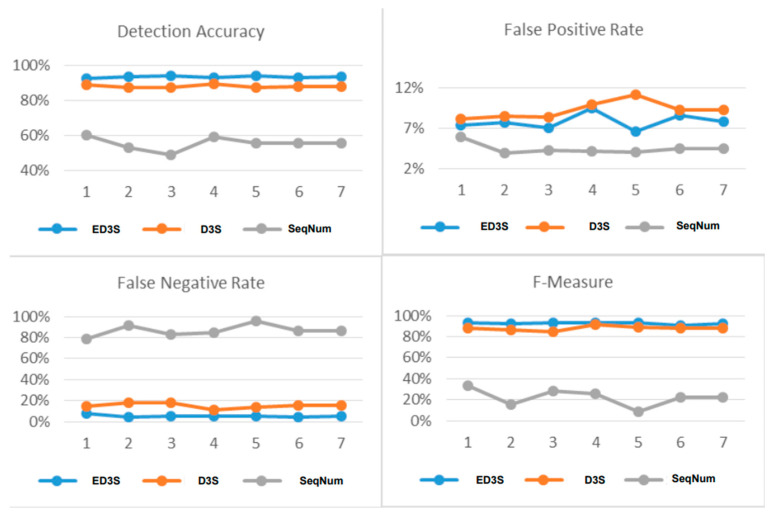
ED3S, D3S, and SeqNum Details Comparison.

Figure 6 displays the proposed scheme’s strength against different de-authentication and dis-association attacks. In Figure 6, the X-axis represents the test number. In contrast, the Y-axis represents the corresponding performance measures in terms of detection accuracy, FPR, FNR, and F-measure.

As shown in thr Figure 6 accuracy section and Table 1, in most tests, ED3S maintained a stable accuracy above 93% for most attack types, compared to D3S whose accuracy dropped below 88%. Meanwhile, the accuracy of the SeqNum scheme slightly fluctuates below 60%. Figure 6 shows that the sequence number’s FPR is more stable than the proposed schemes ED3S and D3S. However, the FNR in Figure 6 false-negative rate part of ED3S and D3S is more stable and much lower than that of the SeqNum scheme. The F-measure part in Figure 6 shows that the overall performance of both ED3S and D3S is stable and around 90%, while it varies below 40% for the SeqNum scheme.

The numerical results of the four evaluation metrics show that the proposed scheme is more robust than the compared schemes in detecting and mitigating DoS attacks. The detected false management frames will not take effect as long as the access point detects them and drops them silently. As a result, the proposed scheme can effectively detect and mitigate false management frames early because it can more accurately provide representative features of both legitimate and fake messages.

The NN is trained on a dataset, and its performance is highly dependent on the quality and representativeness of the training data. Therefore, a potential limitation is that if the training dataset is not diverse enough, or if there are changes in the network or traffic patterns that were not present in the training data, the NN’s performance may decrease. Another potential drawback is the computational complexity of using NN for detection, which can require significant computing resources and time. As a result, the proposed scheme may require high computational resources. Moreover, the proposed scheme is designed specifically to detect management-frames-based DoS attacks and may not be effective in detecting other types of attacks. Therefore, it should be used in conjunction with other security mechanisms and protocols to ensure comprehensive protection against various types of attacks.

In terms of limitations of the model, there may be constraints in its applicability, accuracy, or robustness to certain types of attacks or changes in network behaviour. Furthermore, the model may also be limited by the computational resources required to run the NN, particularly in real-time network monitoring scenarios where quick response times are important.

## 6. Conclusions

Wireless networks face a severe risk of de-authentication/disassociation attacks, and several solutions have been proposed to tackle these threats. However, current research is concentrated on detecting fake frames using sequence numbers. Such a strategy is still open to intrusion from adversaries who know the following sequence number. It is worth noting that predicting the next sequence number is a trivial task for even non-skilled attackers. Therefore, this paper proposes an Enhanced De-authentication/Disassociation Detection (ED3S) Scheme using an artificial neural network as the outcome of this paper. The hypothesis is that the de-authentication/disassociation frames coming from genuine stations can be distinguished from those of the attackers and can be given a feature space. Accordingly, the necessary features were derived from the raw data samples and used to train an artificial neural network model for pattern recognition. The neural network was used to learn the difference between fake and genuine de-authentication/disassociation frames. Performance analysis of the proposed scheme on a semi-realistic dataset shows that the proposed scheme performs well compared to the SeqNum-based scheme. Neural networks can “learn” nonlinear mappings and provide accurate class label prediction from a given sample of data. Applying the suggested model compared to D3S resulted in an overall performance improvement of 4.71. in [15] and 67.06 in comparison to the SeqNum-based scheme. However, the results show that the access point will not execute a total of 7.87% of the genuine messages. This implies that the access point will reserve extra resources for a station no longer interested in the network. However, the proposed scheme still has some limitations that need to be addressed to improve its accuracy and reduce false positives. For future work, we plan to apply an enhanced memetic adaptive method to an on neural network model [23], to improve D3S by increasing the detection accuracy and decreasing the false alarm rate of the original scheme. In contrast, comparing the speed and computational requirements of different algorithms can be a valuable part of future work, as it can help advance the field by identifying the most efficient and effective methods for solving specific problems.

## Figures and Tables

**Table 1:** The Results of the ED3S Scheme, D3S Scheme, and SeqNum Based on Four Scenarios.

Scheme	Scenarios	Accuracy	FPR	FNR	F-SCORE
ED3S Scheme (The Proposed)	1	92.48%	7.33%	7.69%	92.86%
	2	93.57%	7.67%	4.47%	92.02%
	3	93.78%	7.03%	5.14%	92.95%
	4	92.71%	9.46%	5.32%	93.16%
Average		93.14%	7.87%	5.66%	92.75%
D3S Scheme	1	88.87%	8.11%	14.43%	88.02%
	2	86.96%	8.45%	17.81%	86.07%
	3	87.26%	8.37%	18.38%	84.84%
	4	89.30%	9.91%	11.17%	91.25%
Average		88.10%	8.71%	15.45%	87.54%
SeqNum Based (Baseline Scheme)	1	59.91%	5.89%	78.79%	33.18%
	2	52.78%	3.88%	91.16%	15.68%
	3	48.80%	4.20%	83.31%	27.93%
	4	58.86%	4.07%	84.36%	25.98%
Average		55.09%	4.51%	84.40%	25.69%

## Data Availability

Not applicable.
